# A novel marine mesocosm facility to study global warming, water quality, and ocean acidification

**DOI:** 10.1002/ece3.1670

**Published:** 2015-09-30

**Authors:** Gustavo Duarte, Emiliano N. Calderon, Cristiano M. Pereira, Laura F. B. Marangoni, Henrique F. Santos, Raquel S. Peixoto, Adalto Bianchini, Clovis B. Castro

**Affiliations:** ^1^ Instituto Coral VivoRio de JaneiroBrazil; ^2^ Museu NacionalUniversidade Federal do Rio de JaneiroRio de JaneiroBrazil; ^3^ Pós‐Graduação em Oceanografia BiológicaInstituto de OceanografiaUniversidade Federal do Rio GrandeRio GrandeBrazil; ^4^ Instituto de Microbiologia Paulo de GóesUFRJRio de JaneiroBrazil; ^5^ Instituto de Ciências BiológicasUniversidade Federal do Rio GrandeRio GrandeBrazil

**Keywords:** Experimental biology, flow‐through system, marine mesocosm, open‐source board control system, *p*CO_2_ reactor, realism, stress physiology

## Abstract

We describe a completely randomizable flow‐through outdoor mesocosm for climate change and ecotoxicology studies that was built with inexpensive materials. The 16 raceway tanks allow up to 6× water renewal per hour, avoiding changes in natural abiotic seawater conditions. We use an open‐source hardware board (Arduino) that was adapted to control heaters and an innovative CO_2_ injection system. This system reduced seawater pH up to −0.9 units and increased temperature up to +6°C in three treatments and a control. Treatments can be continuously compared with the control and vary according to diel fluctuations, thus following the diel range observed in the sea. The mesocosm facility also includes an integrated secondary system of 48 aquaria for ecotoxicology studies. We validated the reproducibility and relevance of our experimental system by analyzing the variation of the total DNA of the microbial community extracted from corals in three elevated temperature scenarios during a 40‐day experiment. We also present data from temperature, acidification, and copper contamination trials, which allowed continuous, reliable, and consistent treatment manipulations.

## Introduction

Earth faces a period of relatively fast changes in its oceans. Several studies have demonstrated an increase in mortality, diseases, and other physiological changes in marine organisms, especially coral reefs (Bellwood et al. 2004; Bruno and Selig 2007; Stone 2010; De'ath et al. 2012; Hoegh‐Guldberg 2014). Global warming and ocean acidification are among the significant environmental problems facing today's policymakers. The scientific community is expected to provide predictions of the environmental consequences associated with continued atmospheric emissions (Hoegh‐Guldberg 2014). However, forecasting future long‐term effects on the survival and physiology of these organisms is difficult to achieve in field studies. How do these changes affect organisms and ecosystems? What are the critical thresholds for these changes? Will organisms be able to adapt? Given the need to reproduce future conditions, most knowledge must be derived from controlled manipulative experiments. However, few published papers meet these requirements (Stewart et al. 2013).

Among a myriad of experiments, those using mesocosm facilities fill the gap between smaller scale laboratory experiments and field data (Kraufvelin 1999; Benton et al. 2007; Widdicombe et al. 2010; Stewart et al. 2013). We use the concept of a mesocosm as an outdoor experimental setup partially connected to the natural environment (Odum 1984) despite varying definitions of mesocosm in the literature (Stewart et al. 2013). Mesocosms reduce experimental stress on tested organisms, mimic real conditions experienced by organisms in the field, and maintain control of the variables being tested (Widdicombe et al. 2010). The trade‐offs of mesocosms include the difficulty and costs associated with achieving appropriate and statistically sound replicates (Gamble 1990; Heffner et al. 1996).

We developed an outdoor mesocosm system that followed natural diurnal variations in tested and other variables. The system replicated all major physical and chemical factors of the natural environment as much as possible, attaining increased ecological realism and experimental replicability as proposed by Wernberg et al. (2012) and Kraufvelin (1999). The system was designed to achieve the following criteria: (1) Control tanks closely mimic field conditions in key variables (temperature, *p*CO_2_, etc.); (2) random assignment of replicate treatment tanks; (3) strong flow‐through with a high rate of water renewal; (4) light spectrum, irradiance, and photoperiod are as similar as possible to natural conditions; and (5) accessible cost. The following features of our system are unique: (1) Treatment variables are controlled by an open‐source, Arduino‐based system; (2) treatment variables are continuously compared with controls and instantly adjusted to mimic diurnal curves; (3) we developed a CO_2_ water‐mixing chamber that optimizes gas use; (4) we used underground conditioning tanks to minimize interference from the sun heating the water; and (5) approximately all parts are nonmetallic to avoid metal contamination. We describe our methods and the bottlenecks of building the system in detail and present actual responses of key variables (temperature and *p*CO_2_).

## Material and Methods

**Figure 1:**
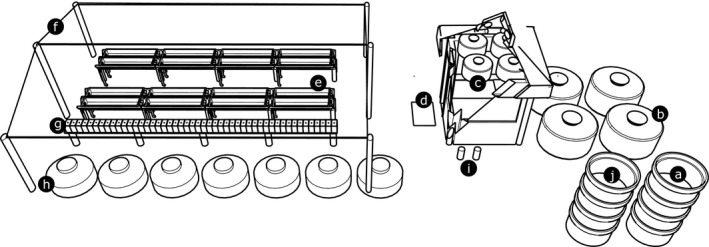
Schematic view of the mesocosm system. Well for intake water pump (a); underground treatment sumps (b); section of control room with four 310‐L reservoirs in the attic (c); randomization hoses (d); raceway tanks of the primary system (e); shade cloth (f); ecotoxicology aquariums of the secondary system (g); ecotoxicology contaminants reservoir (h); carbon filters (i); and water return well (j).

**Figure 2:**
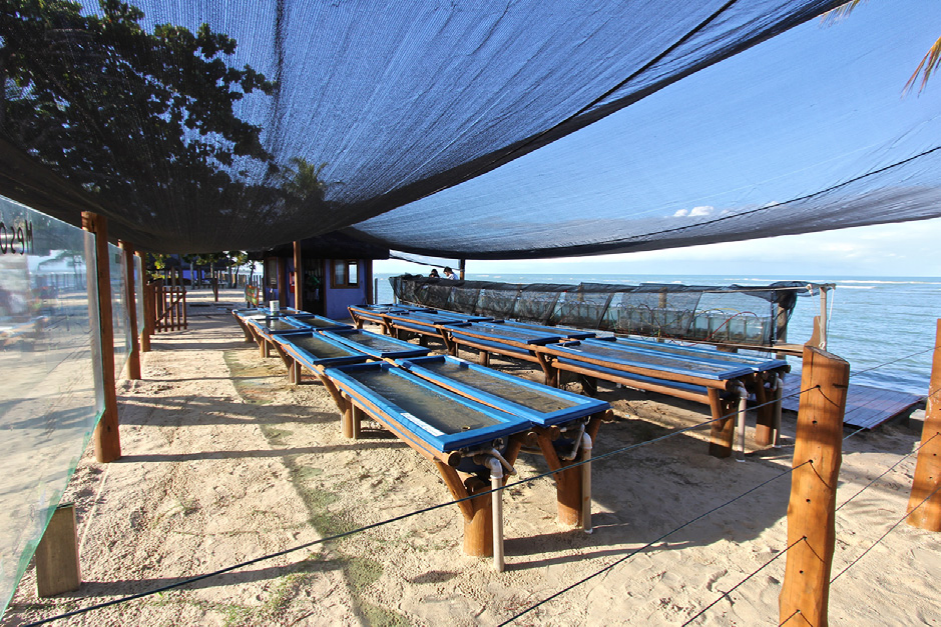
Panoramic view of the mesocosm system as observed from the raceway tanks.

**Figure 3:**
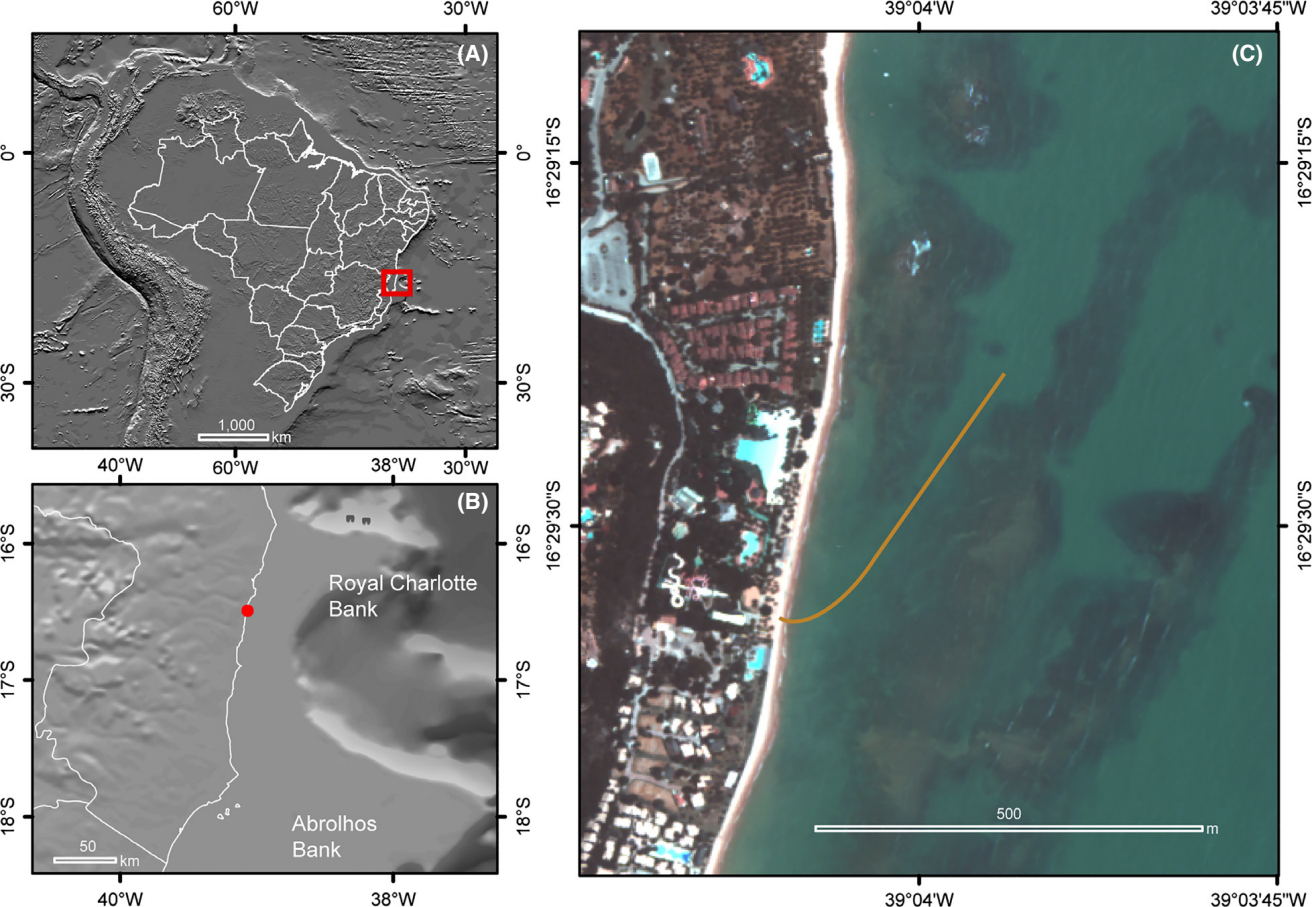
Location of study area (A, B). Mesocosm location and position of the water intake pipe (C).

In 2011–2012, we built a flow‐through mesocosm to study the environmental impacts and stresses on the reef environment in Arraial d'Ajuda District, Porto Seguro, Bahia, Brazil, at the Coral Vivo Project Research Station (Figs. 1, 2, 3). The tanks and aquariums of the mesocosm system receive continuous seawater flow taken from the adjacent reef environment. The mesocosm contains two parallel experimental systems. The first system is used to perform experiments in larger volume tanks (130 L) and simulate climatic stresses, such as higher temperatures and/or acidified seawater, whereas the second system is used to combine these climatic stresses with chemical contaminants, such as metals or nutrients, supplied by an accessory system.

### Water intake and treatment sumps

The water intake facility is close to a fringing reef 500 m offshore (Fig. 3). We used polyvinyl chloride (PVC) pipes (#10121035, inner diameter 91 mm, wall thickness 6.1 mm, working pressure 7.5 kgf·cm^−1^; Tigre, SC, Brazil) for the intake. To avoid clogging or obstruction, the tip was protected with a “T”‐shaped pipe entirely perforated by 8 mm holes, which allow the entry of plankton and particulate organic matter. Each 50‐m PVC pipe section was glued on land using a PVC union (slip × slip) and then attached to each previously installed section underwater after being towed to the installation site by boat. The pipe was attached to the seafloor using *U*‐shaped iron “clamps” fastened to the bottom of every 15 m of tubing with 50 kg concrete weights. On land, the pipe was connected to a hose (inner diameter 62.5 mm, working pressure 6.7 kgf·cm^−1^; Kanaflex KM series, SP, Brazil) and was placed underground at the beach to the pump well.

The water is pumped via a self‐priming water pump (3 HP; Jacuzzi, Itu, SP, Brazil) with a nylon alloy volute and impeller. The pump was installed in a 2.5‐m‐deep well close to the high tide level and pumps at a rate of 0.006 m^3^·s^−1^. The seawater from the pump goes to four 5000‐L underground sumps (Fig. 3) (vertical cistern 5000; Fortlev, Camaçari, BA, Brazil), with a plastic float valve at the sump's intake to maintain a constant water volume. The underground sump installation takes advantage of the thermal inertia of the soil, thereby avoiding insolation, minimizing heat exchange with the atmosphere, and ultimately resulting in a more stable temperature. Up to four treatments (including the control), in combination or alone, can be applied using these sumps (Table 1).

**Table 1:** Seawater temperature during a system trial in 2012 (see Fig. 6). “Reef” refers to temperatures measured at the water intake. “Difference” indicates differences between target and average values with the exception of “Control”, where “difference” indicates the difference between “Reef” and “Control”

	Reef (°C)	Control (°C)	+1 (°C)	+2 (°C)	+4.5 (°C)
Average	26.569	27.141	28.490	29.287	31.495
SE	0.021	0.019	0.020	0.024	0.022
Difference		+0.572	+0.350	+0.146	−0.146
Temp. max.	28.841	28.866	30.318	31.153	33.287
Temp. min.	24.195	24.992	25.234	25.089	25.355

### Control system

An open‐source Arduino (http://arduino.cc) platform hardware board (Reef Angel, Fremont, CA, USA) was adapted to receive extra sensors. The system consists of a control board programmed in C/C++ and has a Creative Commons Attribution Share‐Alike license (CC BY‐SA). The code is available in GitHub for download (https://github.com/mesocosmCV/CoralVivo.git). To protect the control system hardware from the weather and salt splashes, a control room was built.

Four temperature sensors (DS18B20 1‐wire digital thermometer; 12 bits – 0.0625°C resolution; Maxim Integrated, San Jose, CA, USA) were used and programmed to alter treatment conditions according to the average circadian variation of the intake water. Temperature was also monitored with five independent underwater temperature data loggers (Onset Corp., HOBO Water Temp Pro, Bourne, MA, USA). One data logger was located at the water intake on reef, and one unit was located inside each of the four reservoirs installed in the control room attic (Fig. 1).

**Figure 4:**
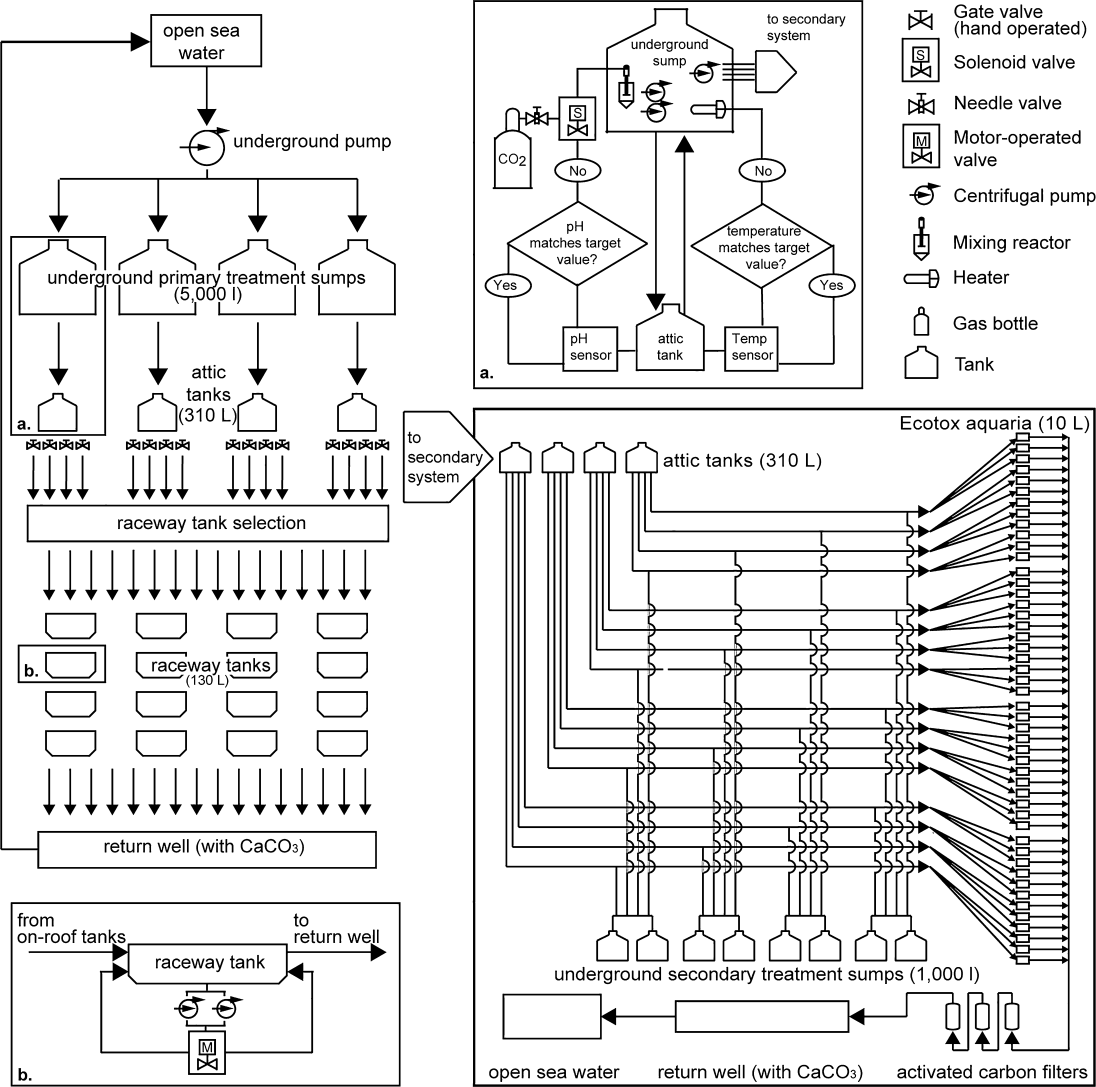
Schematic flowchart of the water flow in the mesocosm system facility showing water intake, the primary system, and the secondary ecotoxicology system; (a) Arduino Reef Angel board logical flowchart, heating and CO_2_ injection system; (b) detail of bidirectional oscillating flow system in raceway tanks.

To manipulate the partial pressure of carbon dioxide (*p*CO_2_), the Reef Angel was adapted to work with three additional pH control modules using a BNC port attached to an extra USB HUB module (Reef Angel) with four pH sensors. We tested two sensors brands, Hanna HI 1090B/5 (Hanna Instruments, Cluj‐Napoca, Jud. Cluj, România) and GEH – 09RBCN Gehaka (Gehaka, São Paulo, SP, Brazil). The Gehaka sensors showed best results with the Reef Angel. The pH sensors were calibrated every day, with pH 7.01 and pH 10.01 standard solutions (Hanna instruments, Woonsocket, RI, USA), through Reef Angel automatic calibration protocol. The sensor's drift and response time was checked weekly using a PG1800 Gehaka benchtop pH meter test protocol (Gehaka). The system controls gas flow to the sumps through three solenoid valves installed on the 9 m^3^ CO_2_ cylinder. When activated, the solenoid valves inject gas directly into the CO_2_ reactors within the sumps. Two circulation pumps homogenize the *p*CO_2_‐enriched water inside the sumps (Fig. 4).

The controller measures the temperature and pH inside four 310‐L tanks on the attic, taking into account the temperature and pH from the intake water (control). The measurement is converted to a moving average of 20 points every 10 s, which serves as a reference that stabilizes the measured results.

### Heating and acidification treatments

The seawater was heated using three 15,000 W heaters made with stainless steel 316i (Anluz, São Paulo, SP, Brazil). The heaters were immersed in each 5000‐L treatment sump tank. In preliminary trials, the heaters could increase the water temperature up to +6°C above the intake temperature with a flow rate of 6× the experimental tank volume. Each heater has six *U*‐shaped electric heating elements 2 m in length. This length ensures a more homogeneous heat distribution over the element's entire surface.

**Figure 5:**
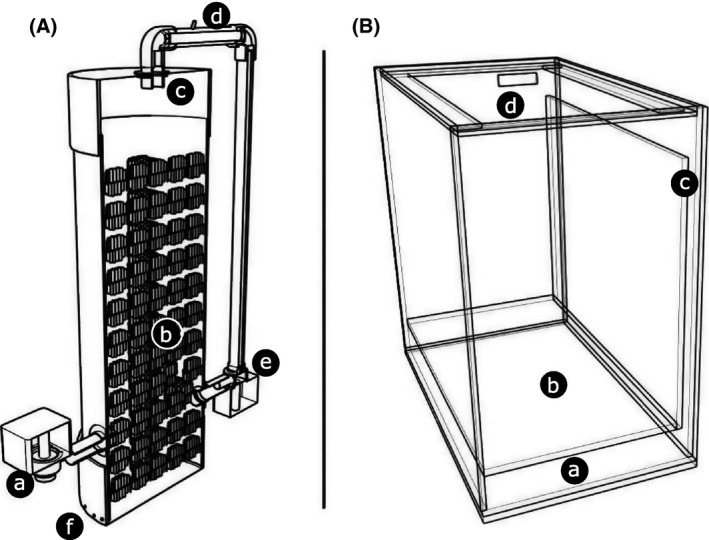
Section of CO_2_ reactor to maximize gas mixing into the water (A). New water enters the reactor through a lateral intake forced by a water pump (a); water flows upward passing by Bioballs^®^ to maximize surface area (b); water is collected in a pipe (c) with a CO_2_ intake (d); water with CO_2_ is “crushed” in a water pump (e), which reduces bubble size and forces the water back into the chamber; larger bubbles move upward re‐entering the “crushing” process, water without large bubbles moves downward, leaving the reactor though small holes at the chamber bottom (f). Secondary system aquarium (B). Built‐in sump (a); sample chamber (b); water overflow (c); and cover lid (d).

*p*CO_2_ was manipulated using a custom‐made CO_2_ reactor system immersed in the sumps (Fig. 5A). The reactor was built from a PVC pipe (200 mm; Tigre) with a dedicated pump that injected a mixture of seawater and CO_2_ into the reactor's intake water, providing a renewal rate of 30 L·min^−1^ and maintaining constant water movement through the reaction chamber. The reactor used a modified CO_2_ bubbling technique (Gattuso and Lavigne 2009; Gattuso et al. 2010; Havenhand et al. 2010). CO_2_ was injected into the water intake of a dedicated pump, forcing the gas bubbles to pass directly through the pump impeller. This action subsequently reduced the size of the gas bubbles and increased the ratio of the bubble surfaces to the reactor volume. The reactor was filled with Bioballs^®^ (Coralife, Franklin, WI) to further enhance the dissolution of gas, producing CO_2_‐enriched water. Each sump tank had two reactors.

Four food‐grade CO_2_ cylinders (9 m^3^; White Martins, Itabuna, BA, Brazil) injected CO_2_ one cylinder at a time through a six‐way needle valve (ISTA CO_2_/Air Metal Flow Regulator, ISTA Products, QR, Tainan City, Taiwan), which allowed a constant and stable flow. Using proper valve adjustment by trial and error and an inverted graduated cylinder filled with water, we established the injection rate that would not over‐acidify the water, even if the control system failed.

### Flow control

Four 310‐L covered water tanks (310; Fortlev) were installed in the control room attic. These tanks receive a continuous flow of water from two serially connected pumps (PL 8000; Via Aqua, Shenzhen, Guangdong, China) installed in each corresponding treatment sump, resulting in a flow of 150 L·min^−1^ between the sumps and the attic tank. The water returns to the sump via gravity through an overflow system installed on top of the attic tanks (Figs. 1, 4). The turnover (29‐fold the attic tanks volume per hour) between the sumps and the attic tanks is considerably higher than the amount of water consumed by the four experimental tanks connected to each attic tank. Thus, the water residence time in the attic tanks was short enough to prevent changes in temperature and pH when compared to underground sumps. The attic tanks remain full of water, and the experimental tanks receive a gravity‐fed steady down‐flow of water. The 1” plumbing allows appropriate flow, which is controlled by adjusting the 16 precision gate valves (PVC 1″; Precision Marine Systems, New Braunfels, TX) (Fig. S1).

### Primary system – experimental tanks

The system has sixteen 130‐L independent polyethylene tanks (Fortelev, Camaçari, BA, Brazil) arranged in a shallow raceway format (Øiestad 1999) with four fully randomizable, interdependent replicates per treatment (Hurlbert 1984) (Figs. 1, 3). A simple randomized hose change determines which tank will receive each treatment in a trial, minimizing method artifacts as proposed by, Hurlbert and White (1993), Underwood and Peterson (1988), and Havenhand et al. (2010).

The water flow in the experimental tanks was adjusted daily to 10 L·min^−1^. The water was measured with a flow meter (TM 050; GPI, Wichita, KS), and a 5× tank turnover rate served as the goal. We therefore ensured that part of the natural diel and seasonal variations of the adjacent reef were maintained in the experimental mesocosm tanks, avoiding changes in water quality, such as variations in salinity due to precipitation (Table S1).

To mimic the reef's water movement, each tank had a 36‐L·min^−1^ bidirectional oscillating flow water circulation system that was built with two Better 2000 pumps (Sarlo Better, São Paulo, SP, Brazil) assembled in parallel and a 15‐s interval motor operated by a two‐way ball valve (NY do Brasil, São Paulo, SP, Brazil) (Fig. 4). This approach promotes increased water oxygenation, reduces the boundary layer of sessile organisms, and enables control of the rate of sedimentation by increasing or reducing the tanks’ hydrodynamics. Samples are placed on acrylic grids on the bottom of the tank. As new water continuously enters the tanks, there is a 1¼″ overflow that leads the water to the tank discard system, which allows the removal of surfactants.

### Secondary system – ecotoxicology aquariums

Forty‐eight 10‐L glass aquariums (20 × 30 × 35 cm) receive treated water from the tanks (control and three treatments) through two centrifugal pumps (Better 2000 PowerHead; Sarlo Better, São Paulo, SP, Brazil) connected in parallel in each attic tank at a flow of 6 L·min^−1^ (Fig. 4) corresponding ~3× aquarium turnover rate. Each experimental aquarium has a “sixth glass” system, allowing a supernatant waterfall that improves oxygenation and surfactant removal (Fig. 5B).

The water is brought to the aquariums via rigid Teflon^®^ 3/8″ hoses with a ball valve at the end of each hose for flow control. To simplify treatment individualization, each set of aquaria receives the same color hose (Fig. S2). This water is mixed with stock solutions (seawater with or without contaminants) in eight 1055‐L underground reservoirs (STP Fortlev 1055) via *in line* dilution using sixteen 0.169‐L·min^−1^ peristaltic pumps (Alifer, São Paulo, SP, Brazil). In metal contaminant trials, each pair of stock solution reservoirs (even with a treatment) is used every other day to allow the metal contaminant stock solution (with desired dilution) to undergo a stabilization process. As a control, natural seawater that has been injected into the system at the same rate as tanks with contaminants is provided to a separate pair of reservoirs. Four ¾″ hoses extend from the attic tanks to feed the aquariums. The configuration and positioning of the aquariums prevents shadows in the samples. To avoid metal contamination, all mesocosm parts (except the *U*‐shaped stainless steel heaters) are made of PVC, silicon, or Teflon. The system may contain up to four dilutions versus four mesocosm treatments combined with three replicates per clustered treatment for a total of 48 experimental tanks. Aquariums have a glass cover to reduce gas loss to the atmosphere. Samples are placed on the aquarium's glass bottom.

### Water discard

Water exits the experimental aquariums and tanks by gravity and flows to an underground PVC tube (100 mm; Tigre, Joinville, SC, Brazil), which collects the used water. Water from the aquaria flows through a three‐step activated carbon filter system consisting of three 40 cm high by 20 cm diameter PVC cylinders, which remove residual copper and other metals (Fig. 4). Water from both systems then enters a pit (1.5 m in diameter and 2.5 m high) that contains CaCO_3_ to neutralize excess acidity. A portion of this water percolates through the sandy bottom of the pit, whereas the remainder is returned to the sea through a PVC pipe (110 mm; Tigre, Joinville, SC, Brazil) that runs 30 m offshore.

### Light intensity

Tanks received only natural sunlight and therefore followed natural day/night light cycles. Sunlight was attenuated with 70% black horticultural shade cloth (30% transmitted light; Sombrite^®^, São Paulo, SP, Brazil), which uniformly reduces light at all visible and nonvisible wavelengths; this would not be possible if the tank system was covered with a glass or polycarbonate roof (Toomey et al. 1995). The mesocosm raceway tanks are aligned in a N‐S direction to ensure homogeneous irradiance for all replicates. Using this approach, the underwater light intensity of the tanks was 479.85 ± 43.8 *μ*mol photons·m^−2^·s^−1^ (average ± SE) (LI‐250 light meter with LI‐190SA sensor; Li‐Cor, NE). This intensity is consistent with the average noon light measured *in situ* (2.5 m depth) at mean tide level in Recife de Fora reef (16°24′31″S; 038°58′39″W) off Porto Seguro, BA.

### Water analysis

Water parameter measurements were made in each of the 16 raceway tanks. Water samples for the determination of dissolved inorganic nutrients (NO_3_ and ${\text{PO}}_{4}^{3-}$) and turbidity were collected every 4 days. The samples were analyzed with a colorimeter (DR/890; Hach, NO). Tanks’ salinity was obtained daily with a hand‐held refractometer (Instrutherm ITREF 10 optical refractometer, SP, Brazil). Dissolved oxygen (DO) was measured daily directly in the raceway tanks, with a portable DO meter with a polarographic sensor (MO900; Instrutherm, São Paulo, SP, Brazil).

Seawater total alkalinity (TA) was measured taking samples (*n* = 4) from underground sumps, at the beginning, in the middle, and at the end of the acidification experiment. Each sample was filtered with a 0.20‐*μ*m mesh filter using a 50‐mL disposable plastic syringe. Samples were then stored in a refrigerator (4°C) in a 250‐mL vial (Duran Borosilicate Glass 3.3; Schott AG, Mainz, Germany). TA analyses were performed within 24 h. However, tests of viable storage times showed no changes in the TA of stored samples after 6 days. TA measures were adapted from Yao and Byrne (1998). Each sample was gently bubbled with N_2_ until all CO_2_ was removed (circa 2 min), as determined in a preliminary test trial. Samples with circa 30 g of water were titrated with 0.11720 (±0.00001) mol·kg^−1^ HCl in a cuvette using a 1‐mL glass syringe (KDS100, KD Scientific, Holliston, MA) fitted to a syringe pump (KD Scientific KDS100). Changes in pH during microtitration were determined at 444, 616, and 750 nm using the colorimetric indicator bromocresol green with a spectrophotometer (Ocean Optics USB4000, Dunedin, FL, USA) and a xenon light source (Ocean Optics PX‐2, Dunedin, FL). The mass of HCl used to reach a pH between 3.9 and 4.2 was used to calculate alkalinity (see Yao and Byrne 1998). The accuracy of determinations of TA was evaluated by analyzing certified reference materials (CRMs) from A. G. Dickson Laboratory, Scripps Institution of Oceanography. CRMs were analyzed showing a mean accuracy of <1.0%. The *p*CO_2_ of each treatment was calculated using CO2Calc version 1.3.0 (Robbins et al. 2010) keying in temperature, salinity, TA, and pH.

For analysis of dissolved copper (Cu) concentration throughout the experiment, water samples (10 mL) of each experimental medium were collected weekly, filtered (0.45‐*μ*m mesh filter), acidified with 1% HNO_3_ (SupraPur; Merck, Billerica, MA), and kept refrigerated until analysis. In the laboratory, water samples were desalted before Cu analysis, as described by Nadella et al. (2009). Dissolved Cu concentration was determined by atomic absorption spectrophotometry with graphite furnace (Perkin‐Elmer, Waltham, MA).

### Biological validation

**Figure 6:**
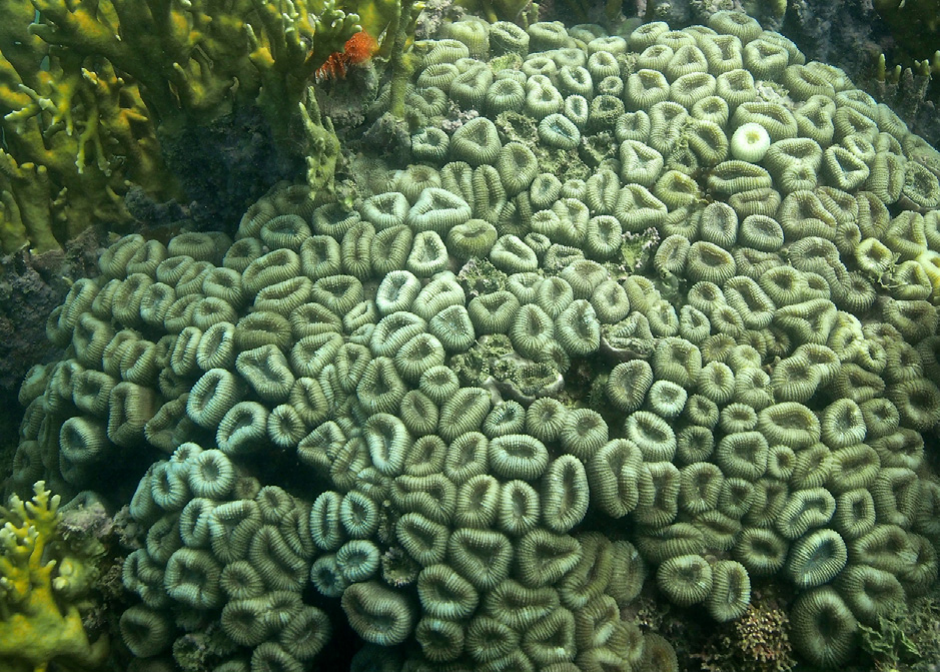
Colony of *Mussismilia harttii* in Recife de Fora reef, Bahia, Brazil.

The operation of the system was validated by determining the bacterial community in the stony coral *Mussismilia harttii* (Fig. 6) under various temperature treatments. Using a mortar and pestle, 0.5 g of dried material from each coral sample was macerated. A total community DNA extraction was performed using the ZR Soil Microbe DNA Kit (Zymo Research, Irvine, CA) (Santos et al. 2012). DNA quality and concentration were evaluated using a Qubit fluorometer (Invitrogen, Carlsbad, CA). The average molecular weight and DNA quality were further assessed using conventional electrophoresis in 0.8% agarose gel with 0.56× TBE buffer (45 mm Tris–borate, 1 mm EDTA, pH 8.0).

The total DNA extracted from all coral samples was subjected to amplification using 16S rRNA gene‐specific primers (L1401 and U968) (Nübel et al. 1999). The amplicons obtained were assessed on agarose gels before DGGE analysis, and the latter was performed with an Ingeny PhorU2 system (Ingeny, Goes, the Netherlands) using a 45–70% denaturing gradient (where 100% denaturant consisted of 7 m urea and 40% formamide) and 6.0% polyacrylamide. Electrophoresis was performed at 60°C at 100 V for 16 h. After electrophoresis, gels were stained with SYBR Gold at a final concentration of 0.5 mg·L^−1^ (Invitrogen, Breda, the Netherlands) and revealed on a UV transilluminator. Gel images were generated using an Image Master VDS (Amersham Biosciences, Buckinghamshire, UK) and stored as TIFF files. The DGGE patterns were compared by clustering the different lanes using Pearson's correlation; the comparisons were performed with GelCompar II software (Applied Maths, Sint‐Martens‐Latem, Belgium) using the unweighted pair‐group method.

## Results and Discussion

The experimental tanks were susceptible to variations in abiotic conditions that were similar to those experienced by the reef, including variations in turbidity, changes in lunar phase and solar irradiance, fluctuating nutrients levels and plankton, varying photoperiods, and other biotic and abiotic factors (Table S1). During precipitation, changes in salinity were not detected (G. Duarte, unpubl. data). A simple change of the shade cloth can be used to simulate irradiance at different reef depths, and UV can be adjusted using a UV filter over the raceway tanks.

**Figure 7:**
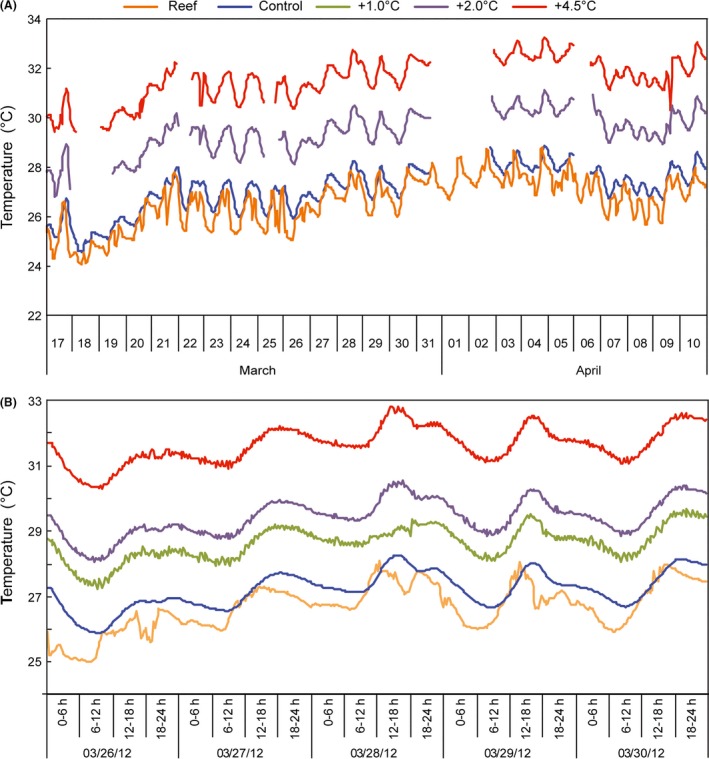
Seawater temperature during a system trial in 2012. (A) Monthly variation in control and treatments. Interrupted lines indicate periods when the heating system was off. (B) Diel variations in control and treatments from 26 March 2012 to 30 March 2012. “Reef” refers to temperatures measured at the water intake.

The system has already been employed to assess changes in temperature, *p*CO_2_, copper contamination, and the combined effects of these variables in pairs or collectively, in four different trials (2012/1, 2012/2, 2013, 2014). The experimental setup described here was improved during these trials. The first temperature trials aimed to raise water temperature by +1, +2, and +4.5°C; the results were similar to the target values (Table 1). The difference between the intake seawater temperature and the control may be considered low, as the first was obtained at a 6 m depth and the second was measured in the system on land. The diel fluctuation in reef water temperature was well‐matched to the control in all treatments (Fig. 7).

**Figure 8:**
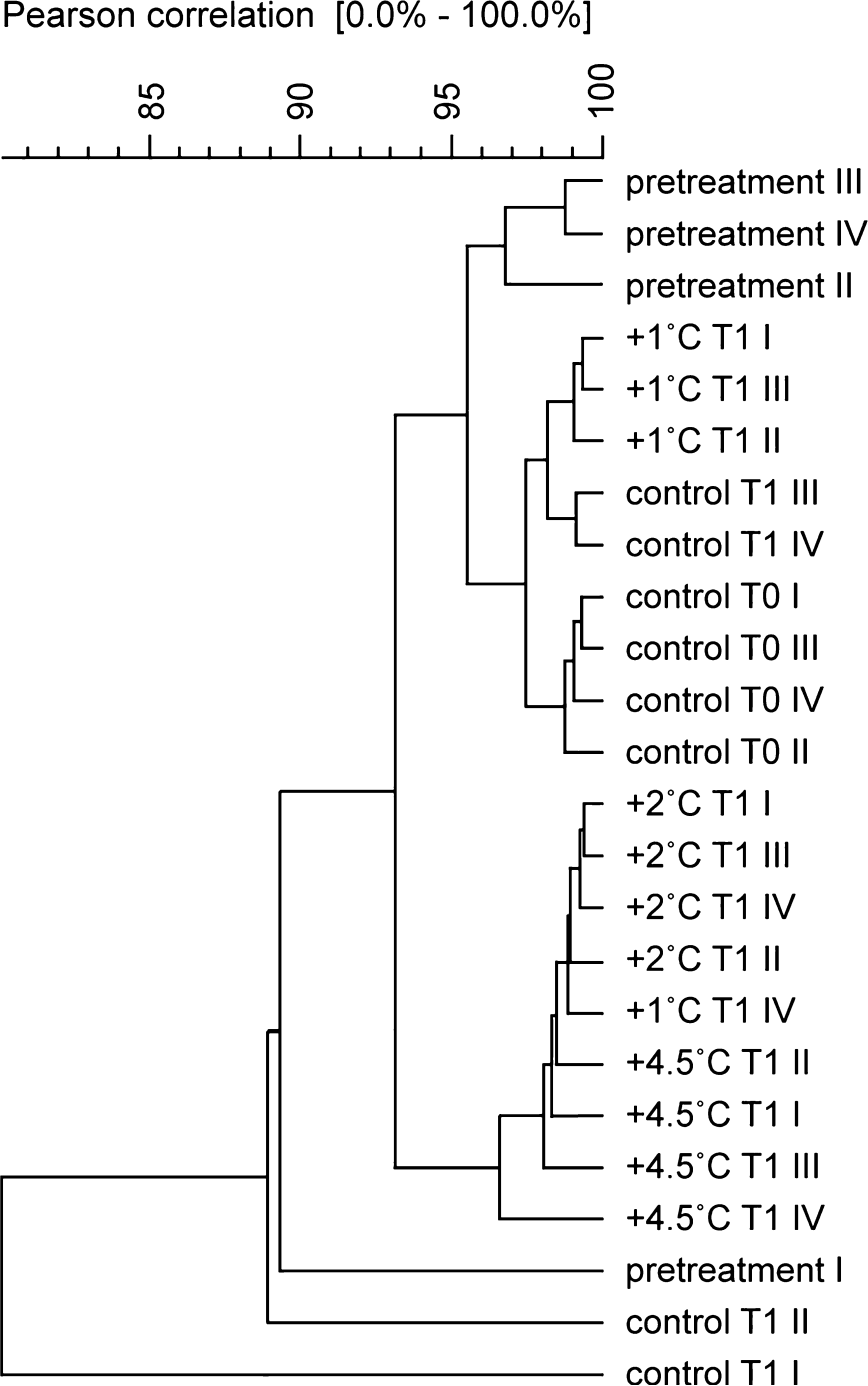
Dendogram of PCR‐DGGE profiles for the 16S rRNA created using UPGMA based on similarities calculated by densitometry. Pretreatment refers to samples collected before the experiment; T0 refers to samples collected after 10 days of acclimatization period; and T1 refers to samples collected after 10 days of temperature treatment.

The results of the biological validation revealed clustering of treatment and control samples (Fig. 8) in the following two groups: one containing most of the control and +1°C samples and the other containing +2 and +4.5°C samples. The precise adjustment of the abiotic variables achieved by the mesocosm in addition to treatments following the diel variation of the controls is observed with the bacterial profile between +1 and +2°C treatments. Although on average these treatments only differed by 0.8°C (Table 1), the difference was sufficient to cluster these treatments into different groups.

An example of the sound performance of our mesocosm system is demonstrated in the first publication produced using this system, which evaluated the effect of realistic increases in ocean water temperature on the photosynthetic quantum yield (*F*_v_/*F*_m_) of the coral *M. harttii* in relation to the abundance and diversity of prokaryotic nitrogen‐fixing bacteria (diazotrophs) associated with this coral. The experiment also revealed changes in the microbial community and maximum quantum yield of photosystem II (*F*_v_/*F*_m_) even among treatments with relatively close temperature differences (+1 and +2°C) (see Santos et al. 2014).

**Figure 9:**
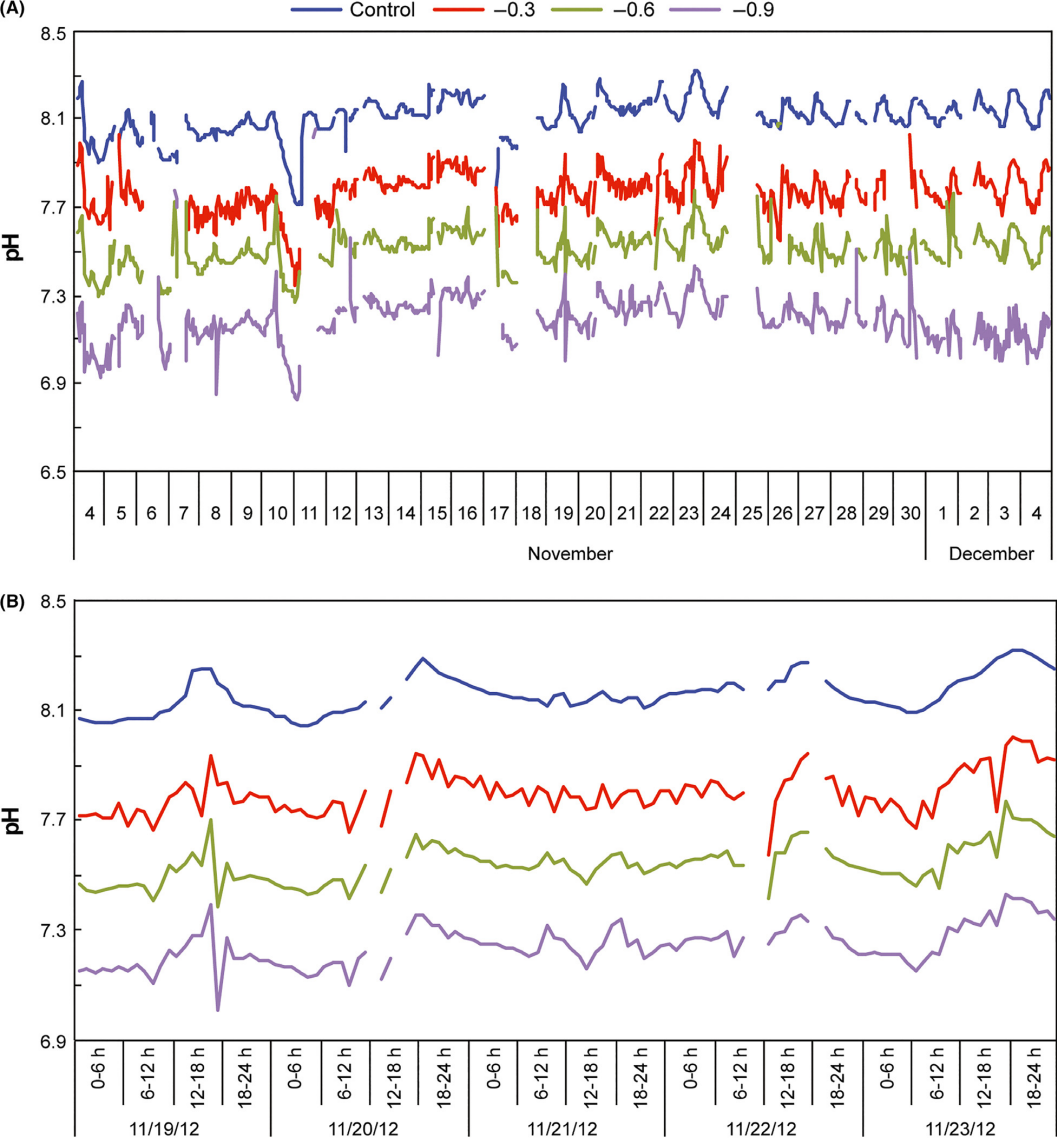
Measurements of pH during a system trial in 2012. (A) Monthly variation in control and treatments. Interrupted lines indicate pH sensor calibration periods. (B) Diel variations in control and treatments, from 19 November 2012 to 23 November 2012.

A *p*CO_2_ trial also exhibited similar fluctuations between the controls and treatments (Fig. 9, Table S2). Minor variations in the −0.3 pH treatment occurred, possibly due to a stronger than desired CO_2_ injection in the reaction chamber when the controller opened the solenoid valve. However, these variations were less than the diel variation in the control, maintaining the individuality of each treatment. A bubble counter device regulated the flow of CO_2_ in this trial. We later found that blowing CO_2_ into an inverted graduated cylinder filled with water and calculating the volume of CO_2_ gas from the volume of water displaced in a minute were more effective; pH fluctuations were reduced using this method. In a combined trial (2014), we regulated the CO_2_ flow differently between the *p*CO_2_ only (80 mL of gas min^−1^) and the *p*CO_2_ plus +4.5°C treatments (90 mL of gas min^−1^) to obtain the same final pH, compensating the influence of temperature in gas loss. Alternatively, a mass flow controller installed in a CO_2_ output could ensure a stable gas stream without manual adjustments. The Reef Angel control system can be improved using four infrared gas analyzers (e.g. Li‐Cor Li‐820) instead of pH probes, providing direct *p*CO_2_ measurements.

**Figure 10:**
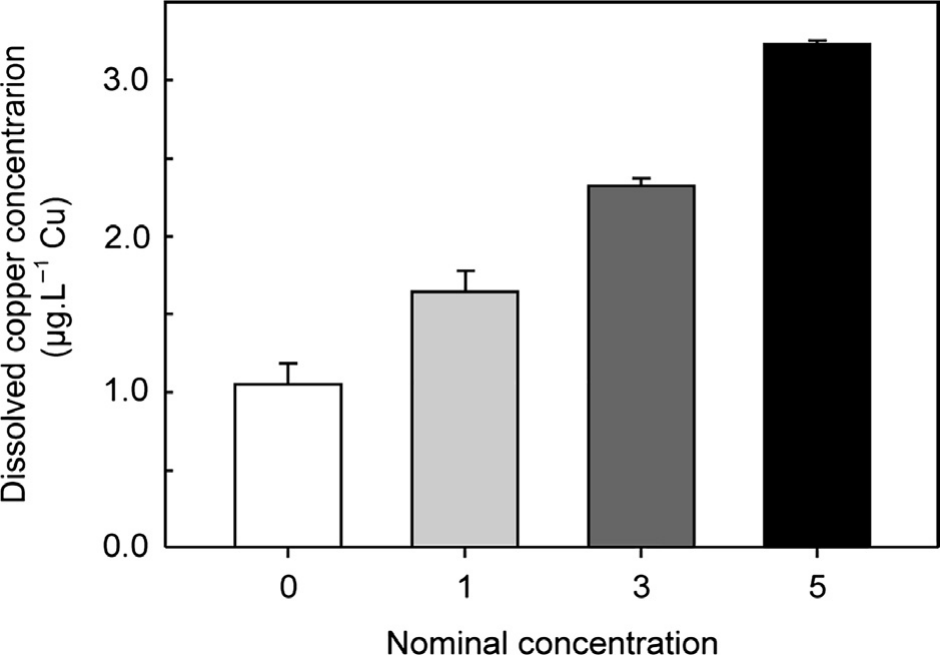
Copper treatment during a 2012 system trial. Bars indicate average concentration between sampling times during the entire experiment (*n* = 3; error bars = standard error).

To determine the minimum differences between the copper concentration that the secondary system could maintain, tests were performed with low concentration differences between treatments. The nominal concentrations tested were 0, 1, 3 and 5 μg/L Cu. Measured copper concentrations (average ± standard error) were as follows: 1.0 ± 0.13 *μ*g·L^−1^ (control; *n* = 4), 1.6 ± 0.12 *μ*g·L^−1^ (*n* = 4), 2.3 ± 0.04 *μ*g·L^−1^ (*n* = 4), and 3.2 ± 0.01 *μ*g·L^−1^ (*n* = 4) (Fig. 10). Many studies use higher concentrations, for example, Martins et al. (2011) and Bielmyer et al. (2010).

The mesocosm facility we developed is a relatively inexpensive, innovative, and ambitious system to deliver significant advances in research regarding the combined effects of water quality, climate change, and ocean acidification. The mesocosm test results allow us to predict and identify without confounding the cause–effect relationships between treatment and control samples (Underwood and Peterson 1988), with repeatability and replicability (Kraufvelin 1999).

Our system allows us to observe and measure the ecological, biological, and physiological stress responses of marine organisms to a variety of water parameters. The system was initially designed for research regarding predicted global change scenarios (temperature and acidification) regardless of the association with copper pollution. However, it is a very versatile system that can be easily adapted to work with a large number of stressors and serve multiple research interests.

## Conflict of Interest

None declared.

## Supporting information


**Figure S1.** Panoramic view of the mesocosm system as observed from the secondary ecotoxicology system aquaria.

**Figure S2.** Inside view of control room, showing the Reef Angel cabinet, the pH sensors, and precision gate valves for flow control.

**Table S1.** Average abiotic parameters concentrations from each treatment of mesocosm raceway tanks (*n* = 4 replicates).
**Table S2.** Summary of values for *p*CO_2_ and Ω_aragonite_ parameters during acidification system experiment.
