# Supplementary figures and images for: A novel marine mesocosm facility to study global warming, water quality, and ocean acidification
Source: Ecol Evol. 2015 Sep 30;5(20):4555–66. doi: 10.1002/ece3.1670 (PMC4670062; doi:10.1002/ece3.1670)

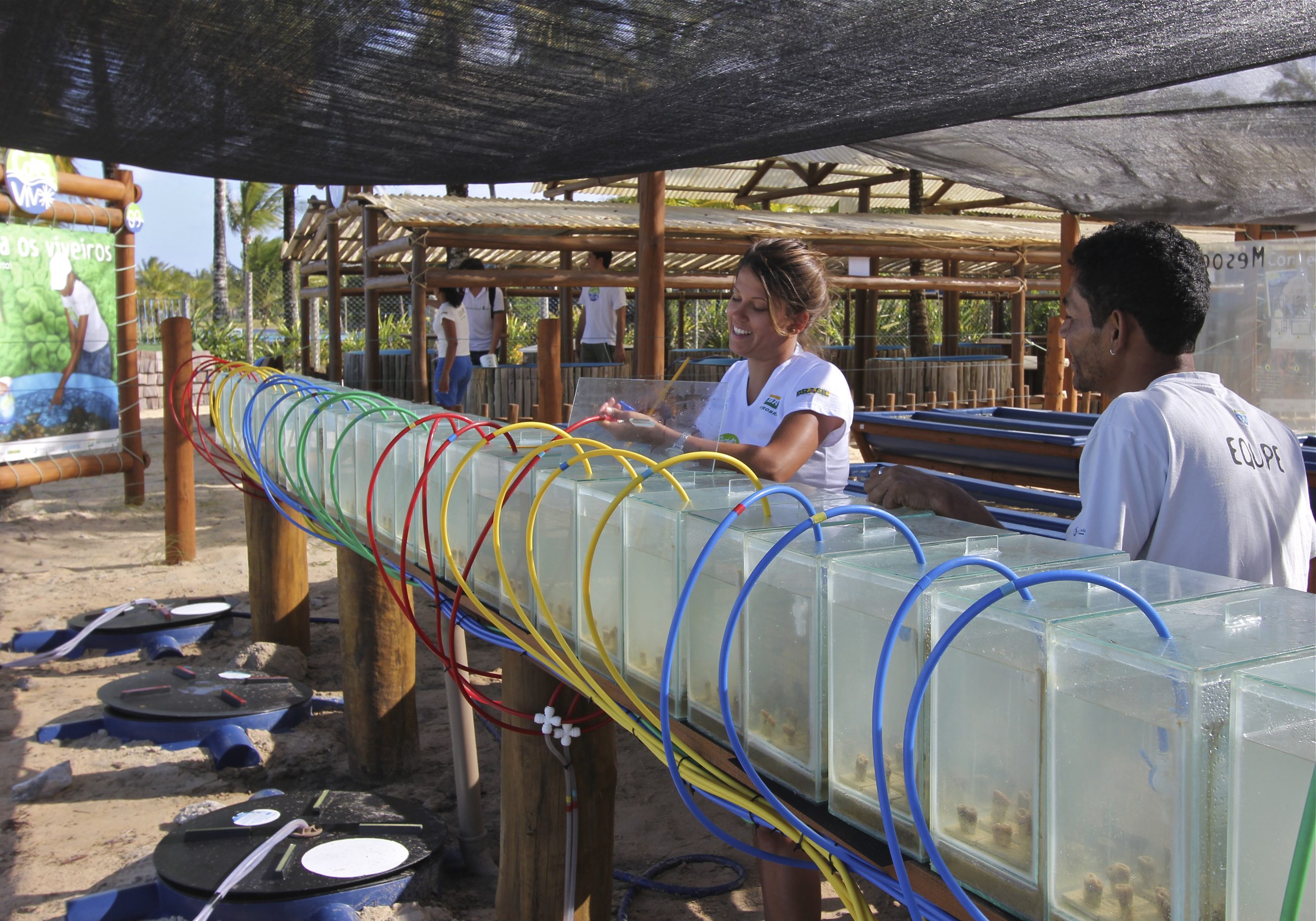

Supplement: Supplementary file 1 — Figure S1. Panoramic view of the mesocosm system as observed from the secondary ecotoxicology system aquaria. [file ECE3-5-4555-s001.tif]

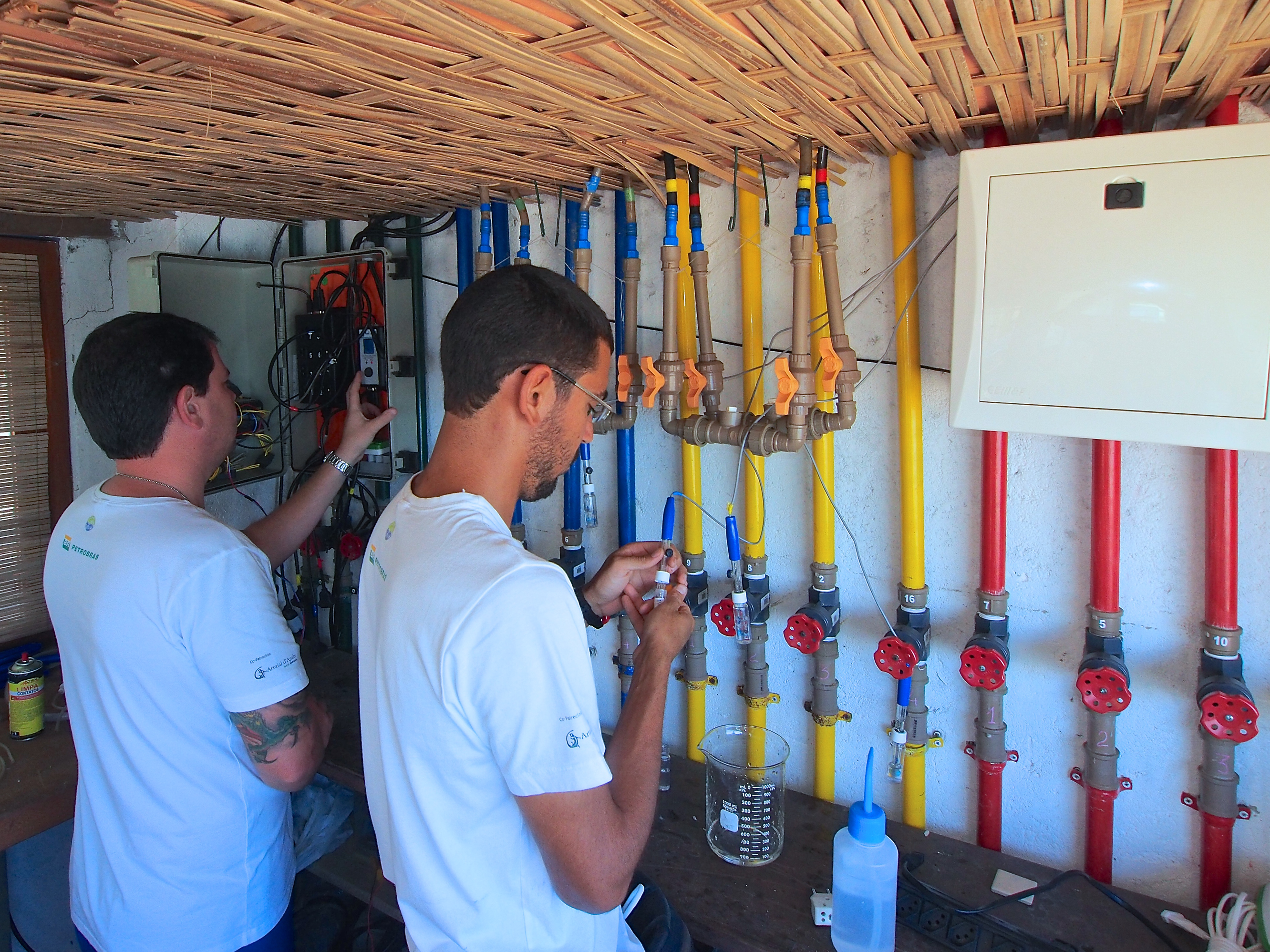

Supplement: Supplementary file 2 — Figure S2. Inside view of control room, showing the Reef Angel cabinet, the pH sensors, and precision gate valves for flow control. [file ECE3-5-4555-s002.tif]
