# Supplementary material for: A novel marine mesocosm facility to study global warming, water quality, and ocean acidification
Source: Ecol Evol. 2015 Sep 30;5(20):4555–66. doi: 10.1002/ece3.1670 (PMC4670062; doi:10.1002/ece3.1670)
Supplement: Supplementary file 3 — Table S1. Average abiotic parameters concentrations from each treatment of mesocosm raceway tanks (n = 4 replicates). Table S2. Summary of values for pCO2 and Ωaragonite parameters during acidification system experiment. [file ECE3-5-4555-s003.docx]

STable I – Average abiotic parameters concentrations from each treatment of mesocosm raceway tanks (n=4 replicates). Salinity (S, ‰; n=124), Dissolved Oxygen (DO, mg.L^-1^, n=80), Nitrate (NO_3_^-^, mg.L^-1^, n=8), Phosphorous (PO_4_^3-^, mg.L^-1^; n=8) and Turbidity (T, FAU; n=8) during an acidification system experiment in 2012 (average (±SE)).

|  | Control | Treatment 1 (pH ‑0.3) | Treatment 2 (pH ‑0.6) | Treatment 3 (pH ‑0.9) |
| --- | --- | --- | --- | --- |
| S | 38.10 (± 3.42) | 38.22 (± 3.43) | 38.15 (± 3.43) | 38.14 (± 3.42) |
| DO | 7.24 (± 0.81) | 6.84 (± 0.76) | 6.73 (± 0.75) | 7.29 (± 0.81) |
| NO_3_^-^ | 0.8 (± 0.30) | 0.83 (± 0.27) | 0.72 (± 0.21) | 0.85 (± 0.60) |
| PO_4_^3-^ | 0.13 (± 0.05) | 0.10 (± 0.03) | 0.13 (± 0.04) | 0.10 (± 0.05) |
| T | 8.57 (± 3.23) | 9.33 (± 3.11) | 8.12 (± 2.87) | 8.50 (± 4.25) |

STable II – Summary of values for *p*CO_2_ and Ω_aragonite_ parameters during acidification system experiment.

| Date | Control | | Treatment 1 (pH -0.3) | | Treatment 2 (pH ‑0.6) | | Treatment 3 (pH ‑0.9) | |
| --- | --- | --- | --- | --- | --- | --- | --- | --- |
|  | *p*CO2 | Ω_aragonite_ | *p*CO2 | Ω_aragonite_ | *p*CO2 | Ω_aragonite_ | *p*CO2 | Ω_aragonite_ |
| 26/10/12 | 296.47 | 5.73 | 754.66 | 3.22 | 1578.11 | 1.83 | 3210.45 | 0.99 |
| 12/11/12 | 422.49 | 4.63 | 1087.35 | 2.42 | 1900.95 | 1.54 | 4119.18 | 0.78 |
| 29/11/12 | 361.95 | 4.90 | 1293.96 | 2.04 | 1899.80 | 1.49 | 4220.99 | 0.73 |
| 30/11/12 | 371.54 | 5.13 | 1322.94 | 2.17 | 1817.94 | 1.64 | 4323.50 | 0.78 |
